# MicroRNA Expression Profile in Peripheral Blood Lymphocytes of Sheep Vaccinated with Nigeria 75/1 Peste Des Petits Ruminants Virus

**DOI:** 10.3390/v11111025

**Published:** 2019-11-05

**Authors:** Yang Yang, Xiaodong Qin, Xuelian Meng, Xueliang Zhu, Xiangle Zhang, Yanmin Li, Zhidong Zhang

**Affiliations:** State Key Laboratory of Veterinary Etiological Biology, Lanzhou Veterinary Research Institute, Chinese Academy of Agriculture Sciences, Xujiaping 1, Lanzhou 730046, China; yangyang01@caas.cn (Y.Y.); qinxiaodong@caas.cn (X.Q.); mengxuelian@caas.cn (X.M.); zhuxueliang@caas.cn (X.Z.); zxll2011@yeah.net (X.Z.); zhangzhidong@caas.cn (Z.Z.)

**Keywords:** miRNAs, PPR, Deep sequencing, PBMC, ST

## Abstract

Peste des petits ruminants (PPR) is one of the highly contagious transboundary viral diseases of small ruminants. Host microRNA (miRNA) expression patterns may change in response to virus infection, and it mainly works as a post-transcriptional moderator in gene expression and affects viral pathogenesis and replication. In this study, the change of miRNA expression profile in peripheral blood lymphocyte (PBMC) from sheep inoculated with PPR vaccine virus in vivo as well as primary sheep testicular (ST) cells inoculated with PPR vaccine virus in vitro were determined via deep sequencing technology. In PBMC cells, 373 and 115 differentially expressed miRNAs (DEmiRNAs) were identified 3 days and 5 days post inoculated (dpi), respectively. While, 575 DEmiRNAs were identified when comparing miRNA profiles on 5 dpi with 3 dpi. Some of the DEmiRNAs were found to change significantly via time-course during PPR vaccine virus inoculated. Similarly, in ST cells, 136 DEmiRNAs were identified at 3 dpi in comparison with mock-inoculation. A total of 12 DEmiRNAs were validated by real-time quantitative PCR (RT-qPCR). The oar-miR-150, oar-miR-370-3p and oar-miR-411b-3p were found common differentially expressed in both PPR vaccine virus-inoculated PBMC cells and ST cells. Targets prediction and functional analysis of the DEmiRNAs uncovered mainly gathering in antigen processing and presentation pathways, protein processing in endoplasmic reticulum pathways and cell adhesion molecules pathways. Our study supplies information about the DEmiRNAs in PPR vaccine virus-inoculated PBMC cells and ST cells, and provides clues for further understanding the function of miRNAs in PPR vaccine virus replication.

## 1. Introduction

MicroRNAs (miRNAs) are highly conserved small, non-coding RNAs of 21–24 nucleotides (nt), which play a critical role in the post-transcriptional regulation by recognizing 3′ end non-coding region (UTR), coding region or 5′ end non-coding region of mRNA [1,2,3]. Studies have implicated that cellular miRNAs could play a regulatory role in immune response to viral infection such as inhibition of antigen processing and presentation by primary macrophages and decreasing the sensitivity of target cells to the antiviral activity of Interferons (IFNs) [1,4,5]. In addition, cellular miRNAs could also significantly influence replication and pathogenesis of viruses such as eastern equine encephalitis virus, hepatitis C virus, human immunodeficiency virus, dengue fever virus, influenza virus and Japanese encephalitis virus [6,7,8,9,10,11,12]. On the other hand, the viral infection also posed an important impact on the expression profiling of cellular miRNA, thereby, regulatory targeting cellular or viral RNA. The emergence and application of miRNA deep sequencing technology has capacitated the direct sequencing of miRNAs, which has been a highly efficient way to study the function of miRNAs [13,14].

Peste des petits ruminants (PPR) is an acute, highly contagious disease of sheep and goats, which remains endemic in Africa, the Middle East and Asia [15,16,17]. The disease is clinically manifested with conjunctivitis, pneumonia, necrotizing and severe pyrexia. The etiological agent, PPR virus (PPRV), is a representative member of genus *Morbillivirus* within family *Paramyxoviridae* [18,19]. PPRV is an enveloped, single-stranded, negative-sense RNA virus with a genome length of 15,948 nucleotides [20,21]. It is now known that PPRV employs the signaling lymphocyte activation molecule (SLAM) expressed on immune cells as a cellular receptor to infect lymphocytes cells, while Nectin 4 expressed on epithelial cells is used by the virus to enter epithelia cells. Members of *morbilliviruses* are immunosuppressive in nature characterized by lymphopenia and cytokine imbalance [22]. An earlier study showed that infection of *morbilliviruses* in peripheral blood mononuclear cells (PBMC) caused suppression of the inflammatory response [23]. Also, the inflammatory and necrotic lesions were observed within epithelial cells-rich tissues in infected animals. However, the mechanisms for this phenomenon are not fully understood yet. PBMCs are the primary targets of PPRV infection in vivo from where the virus reaches different tissue sites [24,25,26]. The recent miRNA expression profiling analysis showed that PPRV infection could elicit significantly up-and down-regulated expression of cellular miRNA in PBMC at 1-day post-inoculation (dpi) in vitro as well as in PBMC, lung and spleen tissues in *vivo*. Target prediction and function analysis indicated that miRNAs might act cooperatively to modulate immune response to PPRV by regulating several immune response genes [27,28,29]. However, the dynamics of cellular miRNA expression profile during PPR vaccine virus inoculation in sheep PBMC in vivo has not been explored up to the current date. In the present study, PBMCs were isolated from sheep inoculated with PPR vaccine virus at 0, 3 and 5 dpi and subjected to miRNA sequencing for examining host miRNAs expression in the immune cells. The primary sheep testicular (ST) cells were widely employed to investigate PPRV pathogenesis associated with epithelial lesions. Therefore, in the present study, ST cells isolated from healthy lambs were also inoculated with PPR vaccine virus in vitro and harvested at 3 dpi for examining host miRNAs expression due to PPR vaccine virus infection in epithelial cells.

## 2. Materials and Methods

### 2.1. Ethics Statement and Animal Experiment

The work was approved by the Animal Ethics Committee of the Lanzhou Veterinary Research Institute (LVRI), Chinese Academy of Agricultural Sciences. Tissues samples and blood samples were collected from sheep according to the good animal practices of the Animal Ethics Procedures and Guidelines of the People’s Republic of China (AEPGPRC). Six healthy sheep (6–12 months old) used in this work, which were negative for PPRV antibody tested by competitive enzyme-linked immunosorbent assay (ELISA) (ID Screen PPR Competition; IDVET, France), were ear-tagged and randomly divided into two groups (uninfected group and infected group). The two group animals were housed in separated rooms in the high-security facility at LVRI. A widely used vaccine strain (Nigeria 75/1 vaccine virus) maintained at LVRI was used as an inoculation virus. Each sheep of the inoculated group was inoculated subcutaneously with a dose (10^4^ 50% tissue culture infectious doses [TCID_50_]/sheep) of Nigeria 75/1 vaccine virus, and each sheep of uninoculated group was inoculated subcutaneously with equal volume of PBS. As PPRV is a lymphotropic virus, the peripheral blood lymphocyte (PBMC) was collected from Nigeria 75/1 vaccine virus inoculated sheep and uninoculated groups (control) using sheep PBMC isolation kit by density gradient centrifugation following the manufacturer’s instructions, respectively. Nigeria 75/1 vaccine virus inoculation was confirmed by RT-qPCR [19].

### 2.2. Primary Sheep Testicular Cells Isolation and Virus Inoculation

Two weeks-old healthy lambs were used to prepare primary testicular cells (ST cells). In short, testicular tissue was collected and washed three times with phosphate-buffered saline (PBS, pH 7.4) containing 100 U/mL penicillin and 100 μg/mL streptomycin. Testicular tissues were cut into small pieces and digested with trypsin at 37 °C for 30 min. Then, PBS and RPMI-1640 (Hyclone, Utah, USA) containing 15% heat-inactivated fetal bovine serum (FBS) (Gibco, New York, USA) were used to wash and resuspend the prepared ST cells, respectively. The ST cells were seeded into cell culture bottles, and then incubated at cell incubator containing 5 % CO_2_ at 37 °C.

For virus inoculation in *vitro*, ST cells were plated at a density of 2 × 10^5^ cells/mL per well in a six-well plate. After the cells grew to roughly 80% confluency, they were washed three times using PBS and then inoculated with Nigeria 75/1 vaccine virus at a multiplicity of infection (MOI) of 0.1. The virus allowed to adsorb for 1 h, followed by washing with PBS and then addition of fresh medium containing 2 % heat inactivated fetal bovine serum (FBS) (Gibco, New York, USA). Uninoculated ST cells were used as the mock-inoculated group. RT-qPCR was used to confirm viral replication in ST cells. Nigeria 75/1 vaccine virus-inoculated and mock-inoculated cells at each time point were taken for three replicates.

### 2.3. Library Construction and Small RNA Sequencing

RNeasy Mini kit (Qiagen, Hilden, German) was used to isolate total RNA from each of the collected samples (PBMC and ST cells). For both PBMC and ST cells, the sequencing libraries were prepared individually for each animal/cell treatment. The integrity and quantity of isolated RNA were assessed with an Agilent2100 Bioanalyzer (Agilent Technologies, Palo Alto, USA) and the NanoDrop ND-2000 spectrophotometer (Thermo Fisher Scientific, Wilmington, USA). The RNA integrity number (RIN) value of all the collected samples was found to be greater than 8, which was suitable for library construction [28]. T4 RNA ligase (Promega, Maryland, USA) was used to ligate the sRNA molecules with a 5′ adaptor and a 3′ adaptor. RT-PCR assay was used to convert the adapter-ligated sRNAs to cDNA according to the Solexa sequencing protocol (Illumina, San Diego, USA). SRNA deep sequencing was carried out in the CapitalBio Technology (Beijing, China). At last, RT-qPCR assay was used to test the miRNAs using the same RNA samples.

### 2.4. Analysis of Small RNA Deep Sequencing Data

miRNAs, tRNAs, rRNAs, small cytoplasmic RNA (scRNAs), small nuclear RNAs (snRNAs), degraded tags from exons and introns and unannotated sRNAs, small nucleolar RNAs (snoRNAs) and signal recognition particle RNAs (srpRNAs) were contained in the sRNA from the deep sequencing data. Firstly, raw reads from each sequencing library were cleaned out by removing the adapter sequences and low-quality tags. The clean reads (18 nt≤ read length ≤30 nt) were screened against Rfam (10.1) database (http://www.sanger.ac.uk/software/Rfam) [28] and the GenBank database (http://www.ncbi.nlm.nih.gov/genbank/) to remove out snoRNA, snRNA, repeats, exon, intron sequences rRNA and tRNA. Then, the remaining clean reads were mapped to the Ovis aries genome. To identify known miRNAs from remaining clean reads, quantifier.pl module was used to align remaining clean reads against miRNA precursor sequences reported in the miRBase database. After except sRNA in the above categories, the unannotated clean reads were put up with novel miRNA forecast employing the miReap program (http://sourceforge.net/projects/mireap/). Differential miRNAs expression profiling between PPR vaccine virus-inoculated and mock-inoculated were carried out applying the DEGseq R package [30]. A *p*-value < 0.01 and a |log2 (fold change)| > 1 were set up as the default thresholds for distinctly differential expression.

### 2.5. Target Prediction of miRNAs

The algorithms of RNAhybrid (http://bibiserv.techfak.uni-bielefeld.de/rnahybrid/) and miRanda (http://www.microrna.org/) were used to predict the target genes for individual DEmiRNAs. Histograms of GO enrichment annotation of the predicted target genes against cell components, biological processes, and molecular functions were created by the WEGO program (http://wego.genomics.org.cn/cgi-bin/wego/index.pl) [31]. The Kyoto Encyclopedia of Genes Genomes (KEGG) pathways gathering analysis were carried out to further probe the biological function of the predicted target genes via the KOBAS 2.0 annotation tool (http://kobas.cbi.pku.edu.cn/help.do).

### 2.6. Validation Using RT-qPCR

To validate the reliability of the deep sequencing data, 12 differentially expressed miRNAs were chosen randomly to conduct real-time RT-qPCR assay. Total RNA from the PBMC and ST cells of control and inoculated were isolated as described above. M-MLV reverse transcriptase (Life, Carlsbad, USA) was used to carry out reverse transcriptase reactions. Power SYBR Green PCR Master Mix (Life) was used to perform real-time RT-qPCR assay on an Mx3005P system (Agilent Technologies) according to the manufacturer’s instructions. The miRNA-specific primers for selected miRNAs are presented in Table 1. The SNORA58 was used as the internal reference gene. Each test was repeated three times. The expression of the selected miRNAs described above was assayed taking the expression of SNORA58 as an internal control. The 2^−ΔΔCT^ method was used to count the relative expression of each miRNA, and the value represented the fold change in the PPR vaccine virus-inoculated group relative to that in the mock-inoculated group [32].

## 3. Results

### 3.1. Confirmation of PPR Vaccine Virus Inoculation in Sheep PBMC and ST Cells

**Figure 1:**
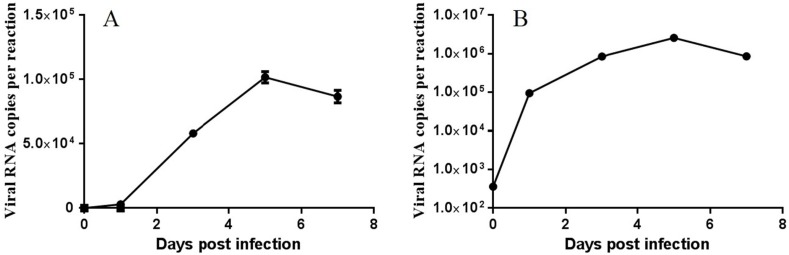
Characterization of peste des petits ruminants (PPR) vaccine virus inoculation in sheep peripheral blood lymphocyte (PBMC) and sheep testicular (ST) cells. (**A**) RT-qPCR analysis of N gene in PPR vaccine virus-inoculated sheep PBMC; (**B**) RT-qPCR analysis of N gene in PPR vaccine virus-inoculated ST cells.

PBMC cells were collected from inoculated sheep with PPR vaccine virus at 0, 1, 3, 5 and 7 dpi, and the viral RNA at each time point were then quantified using RT-qPCR assay. The viral replication curve indicated that the viral RNA in PBMC isolated from inoculated sheep was detected at 1 dpi and then reached the peak at 5 dpi (Figure 1A). Therefore, PBMC isolated from inoculated sheep at 0, 3 and 5 dpi were used for further analysis of miRNA expression. In the ST cells inoculated with PPR vaccine virus in *vitro*, viral RNA was detected by RT-qPCR assay at 1 dpi, and reached the maximum at 5 dpi (Figure 1B).

### 3.2. Construction and Sequencing of sRNA Libraries

The sRNA high-throughput sequencing employing the Illumina system based on sRNA libraries acquired from the PBMC in inoculated sheep with PPR vaccine virus as well as ST cells inoculated with PPR vaccine virus were carried out, and the results were compared with corresponding mock-inoculated groups. Altogether, 15 libraries (9 from PBMC cells and 6 from ST cells) were generated. Each group contained miRNA from 3 parallel libraries.

In PBMC cells collected at different time-points from inoculated sheep, a mean of 12444735, 11,575,474 and 11,636,375 raw reads were obtained from the 3 dpi, 5 dpi and mock-inoculated groups, respectively. After removing adapter sequences, too small sequences, and low-quality sequences, a mean of 11702373, 8,518,928 and 8,524,515 clean reads were identified in the 3 dpi, 5 dpi and mock-inoculated groups, respectively. A mean of 102,837 (mock-inoculated), 99,127 (3 dpi) and 227,601 (5 dpi) unique reads were perfectly mapped to the Ovis aries genome by Simple Object Access Protocol (SOAP), and a mean of 6,384,905 (mock-inoculated), 4,432,649 (3 dpi) and 5,477,852 (5 dpi) total reads were perfectly mapped to the Ovis aries genome by SOAP (Table S1 and File S1).

In ST cells inoculated with PPR vaccine virus in *vitro*, a mean of 13,025,617 and 10,638,071 raw reads were obtained from the inoculated and mock-inoculated groups, respectively. After removing adapter sequences, sequences smaller than 18 nt, and low-quality sequences, a mean of 7,660,261 and 8,181,211 clean reads were identified in the inoculated and mock-inoculated groups, respectively. A mean of 47,124 (mock-inoculated) and 82,627 (inoculated) unique reads were perfectly mapped to the Ovis aries genome by SOAP, and a mean of 6,562,282 (mock-inoculated) and 3,148,946 (inoculated) total reads were perfectly mapped to the Ovis aries genome by SOAP (Table S1 and File S2). Moreover, all the clean reads were annotated and classified as precursor miRNA, mature miRNA, snRNA, intron, exon, and repeats, srpRNAs, snRNAs, rRNAs, snoRNAs and tRNAs. The length of the sRNAs from all the libraries was mainly distributed in 22–24 nt, and is consistent with the representative length of the mature miRNAs [33].

### 3.3. Expression Analysis of miRNA upon PPR Vaccine Virus Inoculation at Different Time Points

**Figure 2:**
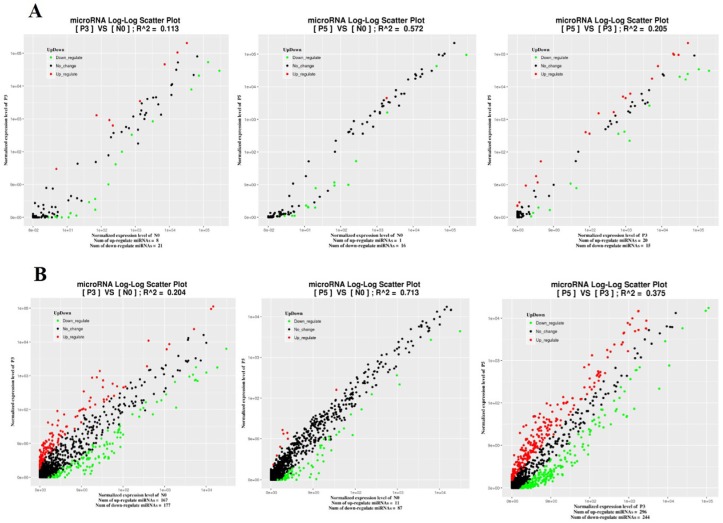
Comparison of expression levels of known miRNAs (**A**) and novel miRNAs (**B**) in PPR vaccine virus-inoculated at 3 dpi (P3) or 5 dpi (P5) and mock-inoculated (N0) sheep PBMC. X and y axes represent the expression levels of the miRNAs of the two groups. The red points represent miRNAs with fold changes greater than 2; the blue points represent miRNAs with fold changes between 0.5 and 2; the green points represent miRNAs with fold changes less than 0.5.

To identify known miRNA in sheep PBMC and ST cells, Nucleotide Basic Local Alignment Search Tool (BLASTN) searches were used to align the small RNA from our libraries to the known mature miRNA and their precursors of the reference species (Ovis aries) in the Sanger miRBase database to obtain the miRNA count as well as the base bias at the first position. Employing a fold change (log 2 FC) ≥ 1 and *p* value ≤ 0.05 as the cut-off value, the total numbers of the miRNA changed after PPR vaccine virus inoculation in PBMC and ST at different time points are presented in Table 2. A total of 373 miRNAs (175 up-regulated and 198 down-regulated) were dysregulated in the PBMC of PPR vaccine virus inoculated sheep at 3 dpi compared with 0 dpi, and 115 miRNAs (12 up-regulated and 103 down-regulated) were dysregulated at 5 dpi compared with 0 dpi, and 575 miRNAs (316 up-regulated and 259 down-regulated) were dysregulated at 5 dpi compared with 3 dpi (Figure 2 and File S1). Among these dysregulated miRNAs, some were up-regulated upon PPR vaccine virus inoculation to 3 dpi, while down-regulated between 3 dpi to 5 dpi, such as 11_3597-3p, 11_3894-5p and 11_4098-5p (File S1). In contrary, some were down-regulated upon PPR vaccine virus inoculation to 3 dpi, while up-regulated between 3 dpi to 5 dpi, such as 10_2877-3p, 12_5448-3p and 13_6176-5p (File S1). The 12_5813-5p (a novel miRNA) was the only miRNA in which expression was constantly down-regulated by PPR vaccine virus inoculation at different time points, while there was no miRNA in which expression was constantly up-regulated at different time points (File S1). This result suggested that miRNA might play important role in PPR vaccine virus inoculation.

**Figure 3:**
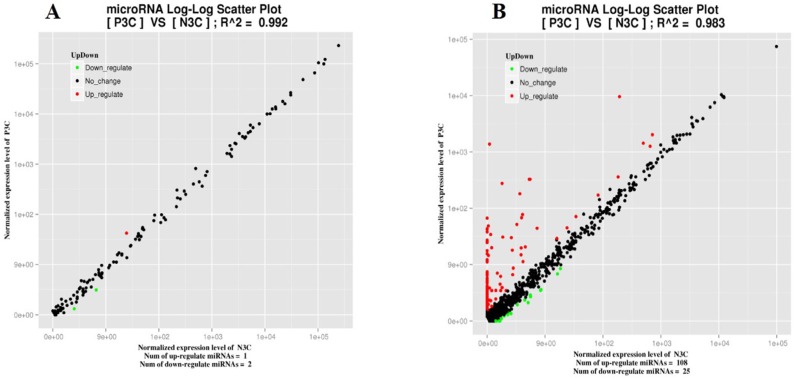
Comparison of expression levels of known miRNAs (**A**) and novel miRNAs (**B**) in PPR vaccine virus-inoculated at 3 dpi (P3C) and mock-inoculated (N3C) sheep ST cells. X and y axes represent the expression levels of the miRNAs of the two groups. The red points represent miRNAs with fold changes greater than 2; the blue points represent miRNAs with fold changes between 0.5 and 2; the green points represent miRNAs with fold changes less than 0.5.

A relatively small number of DEmiRNAs (109 up-regulated and 27 down-regulated) were identified in PPR vaccine virus inoculated ST cells (Figure 3 and File S2). Among these dysregulated miRNAs, 13_9537-5p, 18_14939-5p, 21_21004-5p and 8_36684-3p were significantly up-regulated by PPR vaccine virus inoculation. When it was compared with different tissues of sheep, miR-150, oar-miR-370-3p and oar-miR-411b-3p were found as commonly differentially expressed in PPR vaccine virus inoculated PBMC and ST cells.

### 3.4. Identification of Time Point Specific Different Expression miRNAs

**Figure 4:**
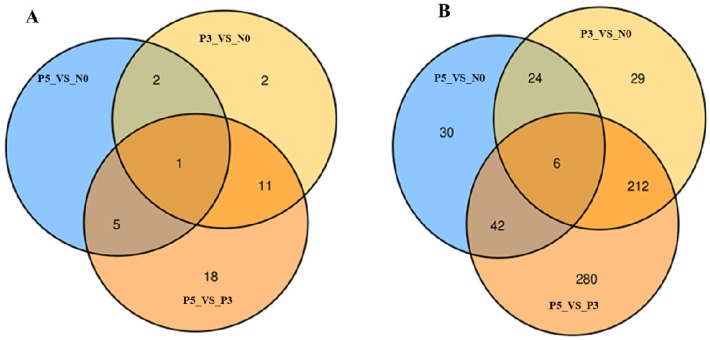
Identification of time point specific different expression miRNAs in PPR vaccine virus inoculated PBMC. Venn diagram displaying the total number of identified time point specific different expression (**A**) known miRNAs and (**B**) novel miRNAs. P3_VS_N0 represents PPR vaccine virus inoculated PBMC at 3 dpi compared with 0 dpi, P5_VS_N0 represents PPR vaccine virus inoculated PBMC at 5 dpi compared with 0 dpi, and P5_VS_P3 represents PPR vaccine virus inoculated PBMC at 5 dpi compared with 3 dpi.

We screened different expression miRNAs which were significantly changed via time-course. For example, a different expression miRNA was identified at 3 dpi compared with 0 dpi but should not change in 5 dpi compared with 0 dpi. Based on this method, we found that 31 different expression miRNAs were specific at 3 dpi compared with 0 dpi (2 known miRNAs and 29 novel miRNAs), 33 different expression miRNAs were specific at 5 dpi compared with 0 dpi (3 known miRNAs and 30 novel miRNAs), and 298 different expression miRNAs were specific at 5 dpi compared with 3 dpi (18 known miRNAs and 280 novel miRNAs) (Figure 4 and File S1). For example, oar-miR-495-3p was 31 fold down regulation at 3 dpi compared with 0 dpi but they were not changed at 5 dpi compared with 0 dpi, and oar-miR-370-3p and oar-miR-411a-3p were 20 fold down regulation and 18.5 fold down regulation, respectively, at 5 dpi compared with 0 dpi but they were not changed at 3 dpi compared with 0 dpi (File S1). On the contrary, there was no change for oar-miR-369-3p and oar-miR-218a at 3 dpi compared with 0 dpi but they were 45 folds up regulation and 9 folds up regulation, respectively, at 5 dpi compared with 3 dpi (File S1).

### 3.5. Target Gene Prediction, and GO and KEGG Analyses

**Figure 5:**
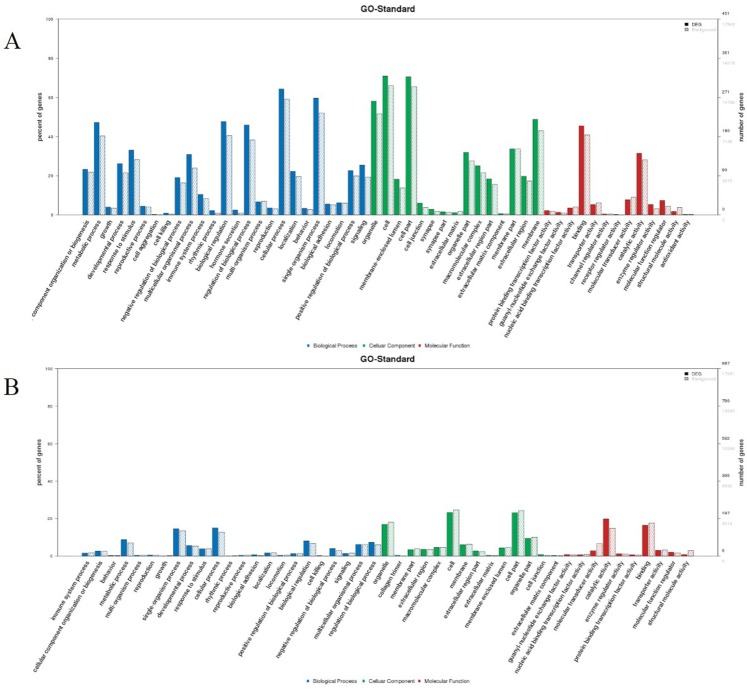
Gene ontology (GO) functional classification of all differentially expressed genes (DEG) predicated from the known miRNA. (**A**) The GO distribution of the DEG in the PPR vaccine virus-inoculated sheep PBMC at 3 dpi versus mock-inoculated; (**B**) The GO distribution of the DEG in the PPR vaccine virus-inoculated ST cells versus mock-inoculated.

To investigate the biological functions of identified differentially expressed miRNAs, two independent algorithms (miRanda and RNAhybrid) were applied to predict each significant differentially expressed miRNAs targeting mRNA. Also, 674 and 6598 target genes for 29 known miRNAs and 344 novel miRNAs, respectively, were predicted as potential miRNA targets in the PBMC of PPR vaccine virus inoculated sheep at 3 dpi compared with 0 dpi (File S3). A GO annotation of the predicted target genes revealed that 4981 target genes were annotated successfully for the 373 miRNAs, and they were involved in endomembrane system, membrane-bounded organelle, regulation of system process and cytoplasmic part (Figure 5A, File S5). The KEGG Orthology Based Annotation System (KOBAS) was used to assign the putative miRNA targets to the Kyoto Encyclopedia of Genes and Genomes (KEGG) pathways for understanding the function of these miRNAs in regulatory networks. KEGG pathway annotation revealed that most of the abundant KEGG terms were involved in biological processes such as antigen processing and presentation, protein processing in endoplasmic reticulum, cell adhesion molecules, miRNAs in cancer, fatty acid metabolism and small cell lung cancer (File S7). Thus, 920 and 2504 target genes for 17 known miRNAs and 98 novel miRNAs, respectively (File S3), were predicted as potential miRNA targets in the PBMC of PPR vaccine virus inoculated sheep at 5 dpi compared with 0 dpi. A GO annotation of the predicted target genes revealed that 5647 target genes were annotated successfully for the 115 miRNAs, and they were involved in intracellular membrane-bounded organelle, membrane-bounded vesicle, single-organism metabolic process, extracellular exosome, hydrolase activity and ion binding (File S5). KEGG pathway annotation revealed that most of the abundant KEGG terms were involved in biological processes such as phagosome, antigen processing and presentation, cell adhesion molecules, fatty acid elongation, phenylalanine metabolism and protein processing in endoplasmic reticulum (File S7).

Putative target genes, 1436 and 24,707 for 3 known miRNAs and 133 novel miRNAs, respectively, were identified in the PPR vaccine virus inoculated ST cells in vitro (File S4). A GO annotation of the predicted target genes revealed that 4090 target genes were annotated successfully for 136 miRNAs and they were involved in peptidase activity, hydrolase activity, cargo receptor activity and vesicle organization (Figure 5B and File S6). KEGG pathway annotation revealed that most of the abundant KEGG terms were involved in biological processes such as lysosome and protein processing in endoplasmic reticulum (File S8). Our results indicate that the differentially expressed miRNAs could regulate virus-host interaction importantly.

### 3.6. Validation of Known and Novel miRNAs by Stem-Loop RT-qPCR

**Figure 6:**
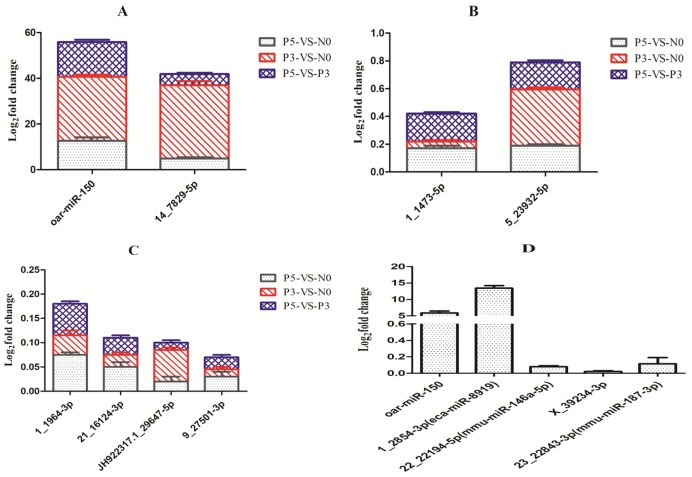
RT-qPCR validate of sequencing data. The change in the miRNA expression of (**A**) oar-miR-150 and 14_7829-5p in PBMC, (**B**) 1_1473-5p and 5_23932-5p in PBMC, (**C**) 1_1964-3p, 21_16124-3p, JH922317.1_29647-5p and 9_27501-3p in PBMC, and (**D**) oar-miR-150, 1_2854-3p, 22_22194-5p, X_39234-3p and 23_22843-3p in ST cells were calculated with SNORA58 as reference gene for normalization. Fold change is represented as log2FC.

Stem-loop RT-qPCR is a robust and extensively applied tool for the test and quantification of mature miRNAs [34,35,36]. In order to verify the expression of the known and novel candidate miRNAs described above, 12 miRNAs were selected randomly to perform stem-loop RT-qPCR using miRNAs from the PPR vaccine virus-inoculated samples and the mock-inoculated samples. Especially, 1 miRNAs (oar-miR-150) commonly expressing in both the PBMC and ST samples were detected, and 7 miRNAs (14_7829-5p, 1_1473-5p, 1_1964-3p, 21_16124-3p, 5_23932-5p, JH922317.1_29647-5p and 9_27501-3p) were specifically observed only in the PBMC sample, and the other 4 miRNAs (1_2854-3p, 22_22194-5p, X_39234-3p and 23_22843-3p) were specifically observed only in the ST sample (Figure 6). The expression of all 12 miRNAs in PPR vaccine virus-inoculated samples and the mock-inoculated samples were confirmed by our experiment, thus proving the reliability of sRNA high-throughput sequencing and computational analysis.

## 4. Discussion

PPR, which causes a serious disease in sheep and goats, is a considerable threat to small ruminants in the world [37]. Deep sequencing technology is generally used to determine DEmiRNA as it is a powerful tool under conditions of physiological perturbation, especially for the low abundance miRNAs [38,39,40]. The miRNAs encoded by a host have been proved to play a critical role in regulating the interaction between virus and host, and their expression and regulation was usually influenced by viral infection [41,42,43,44]. The replication of a virus could be inhibited by some miRNA via targeting viruses or modulating host immune system, while it also could be elevated by other miRNA via regulating the environment in the host [45,46]. Although it has been demonstrated that PPRV infection could induce miRNA expression profiling [27,28,29], time-course miRNA expression profiling in sheep PBMC during PPR vaccine virus inoculation in vivo and ST cells inoculated with PPR vaccine virus in vitro have not been reported yet. This study showed more dynamic information of miRNA expression upon PPR vaccine virus inoculation via time-course. Furthermore, only few studies have reported about miRNA expression profiling against PPRV infection using in vivo and in vitro infection model [27,28,29]. Both PBMC and ST cells were usually used as model cells to investigate the host response during PPRV infection as PPRV is both lymphotropic and epitheliotropic in nature [47,48]. Thus, different infection model for PBMC and ST cells were used in this study to explore the role of miRNAs in modulating the host immune response during PPR vaccine virus inoculation. The potential effect of miRNA expression profiling response in vivo inoculation model might be more complicated than in vitro inoculation model, such as, the linear correlation of miRNA for ST cells was much higher than PBMC cells, while there were few DEmiRNAs for PBMC cells than ST cells at 3 dpi.

In this study, firstly differentially expressed miRNA in sheep PBMC and ST cells inoculated with PPR vaccine virus were determined. Significant replication was shown in both sheep PBMC and ST cells inoculated with PPR vaccine virus at 1 dpi compared with mock-inoculated groups using RT-qPCR. Detailed analysis revealed host miRNAs expression in PBMC and ST cell were changed during PPR vaccine virus inoculation. Among these DEmiRNAs, there were both unique and common miRNA landscapes in different kinds of samples. The oar-miR-150, oar-miR-370-3p and oar-miR-411b-3p were the commonly known miRNAs both expressing in sheep PBMC cells and ST cells. Previous reports showed that the miR-150 could degrade or block mRNA translation through binding to the 3’-untranslated region of target gene [49]. The miR-150 also promoted sheep ovarian granulosa cells apoptosis through inhibition of steroidogenic acute regulatory (STAR) protein expression [50]. The miR-370 inhibited tumor growth with decreased expression in non-small cell lung cancer, while it was found to be oncogene with overexpression in prostate cancer [51]. In our research, some of the known identified DEmiRNAs involved in immune mechanism were found to be unique in PBMC but not in ST cells, such as oar-miR-21, oar-miR-17, oar-miR-379-5p, oar-miR-30a-3p, oar-miR-30a-5p and let-7 family. The let-7 family was found to be highly conserved in sequence and function across species, and it was also related with immunity, specifically with the regulation of immune effectors and toll-like receptors that mediate cytokine expression during pathogen infection [52]. The restoration of let-7 levels might represent a novel therapeutic approach preventing upregulation of some key proteins involved in fibrosis, vascular adhesion and inflammation which were crucial pathological hallmarks of the atherosclerotic process [53]. The miR-21 was one of the first miRNAs identified as an oncogene, and it has been reported as being up-regulated in viral infections and tumors [54]. Hepatitis C virus (HCV) infection results decreased IFN response by up-regulating the expression of miR-21 [55], which promoted viral replication during dengue virus or HIV infection in human cancer cells [56,57]. The expression of miR-21 induced by Epstein-Barr virus (EBV) could promote tumorigenesis through activating the PI3K-Akt pathway which causes FOXO3a to stop repressing miR-21 [58,59]. In addition, an emerging report has proposed miR-21 as an indicator of potential treatment target and disease progression in a mouse model [60]. Furthermore, the downregulation of miR-21-5p might play role in increasing IFN γ production during PPRV infection [29]. The miR-17 has been reported to bind the BVDV genome leading to changes in the host transcriptome [61]. The oar-miR-379-5p and oar-miR-30a might play key roles in apoptosis [62] and PPRV was also reported to cause apoptosis of host cells [63].

On comparing this PPR vaccine virus inoculated PBMC miRNA profile with virulent virus infected PBMC miRNA profile reported in earlier research [29], we found there were small number of DEmiRNAs (42 down-regulated and 26 up-regulated) in PPR virulent virus infected PBMC. Many factors might affect the number of DEmiRNAs in PBMC, such as inoculated with PPR vaccine virus used in our study and infected with PPR virulent virus used in previous report, or acute state of PPR infection model used in virulent virus infected PBMC and PPR vaccine virus which does not clinically infect the animals used in our study. The miR-181a, miR-200c, miR-30 and let-7 family were commonly differentially expressed in PBMC cells among the PPR vaccine virus inoculation and virulent virus infection. Of the 4 DEmiRNAs common in PBMC cells between PPR vaccine virus inoculation and virulent virus infection, miR-181a, miR-200c, miR-30 were found to be down-regulated in both. In addition, let-7 family was found to be down-regulated in PPR virulent virus infection but up-regulated in PPR vaccine virus inoculation. The miR-181a which was a highly conserved miRNA across most vertebrates could play main roles in regulating development, cellular growth, apoptosis, cellular invasion, and tumor suppression [64]. In addition, miR-181a could promote mouse GC apoptosis via targeting SIRT1 [65]. The miR-181a also has been shown to facilitate cardiomyocyte apoptosis by suppressing Bcl-2 expression [66]. Moreover, miR-181a played anti-inflammatory role via repression of the ERK signaling pathway in mice with intervertebral disc degeneration [67]. T cell responses were impaired during HCV infection by miR-181a-mediated expression of DUSP6 which was a marker of T cell aging [68]. The miR-200c which belonged to the miR-200 family was one of the most researched examples in functional miRNAs [69]. The research has showed that tumor suppressors were the main role of miR-200 family members in cancer [70]. In addition, HBV replication was repressed by miR-200c via negatively modulating the expression of NFIA [71]. The miR-200c could also inhibit the expression of PD-L1 and restore the dysfunction of CD8 + T cells, and the role of miR-200c might be antagonized through reactivating SALL4 via STAT3 pathway during HBV infection [72]. Integrative analysis of miRNA expression profile and proteome expression profile showed that identified DEmiRNAs in PPRV virulent virus infected PBMC were instrumental in regulating immune response [29], while our results showed that identified DEmiRNAs in PPRV vaccine virus inoculated PBMC were gathering in antigen processing and presentation pathways, protein processing in endoplasmic reticulum pathways and cell adhesion molecules pathways.

Time-course analysis showed that many miRNAs expressed on PBMC cells at different time points during PPR vaccine virus inoculation were quite similar. These results implied that miRNAs in the normal levels might be important for sheep. However, 373 miRNAs were dysregulated at 3 dpi compared with 0 dpi, and 115 miRNAs were dysregulated at 5 dpi compared with 0 dpi, and 575 miRNAs were dysregulated at 5 dpi compared with 3 dpi. The dysregulation of miRNA might be caused by the exogenous stresses or antiviral immune responses. The up-regulation or down-regulation of these DEmiRNAs could also change at different time of PPR vaccine virus inoculation. For example, hsa-miR-18a-3p, hsa-miR-25-3p, 11_3894-5p and 11_4098-5p were up-regulated from 0 dpi to 3 dpi and down-regulated from 3 dpi to 5 dpi, while hsa-miR-19a-3p, hsa-miR-1246, 12_5448-3p and 13_6176-5p were down-regulated from 0 dpi to 3 dpi and up-regulated from 3 dpi to 5 dpi. These results suggested that miRNA might play a different role in different periods of PPR vaccine virus inoculation in sheep. Previous researches showed that the hsa-miR-18a-3p might work as a master miRNA to control the whole p53-mediated competing endogenous RNA Network [73], and the hsa-miR-25-3p could target the transcription factor TEF-1 to block it by binding with Il-1β, Ccl2, and Ccl3 [74]. The broadly conserved miR-19a-3p was related to cancer progression, and repression of miR-19a-3p could inhibit cell proliferation and enhance cell death [75]. In addition, miR-19a-3p inhibited autophagy-mediated fibrogenesis by targeting TGF-β R II [76]. The miR-1246 played a crucial role in regulation of NF-κB signaling, which increased pro-inflammatory responses in different cell types. Furthermore, the secretion of chemokines and pro-inflammatory cytokines IL-6, CCL2 and CCL5 in MSCs induced by miR-1246 was mediated via NF-κB, but independent of TNFα [77].

The expression of multiple mRNA might be regulated by each miRNA, and the host responses might also be influenced by miRNA expression during viral infection [10,78,79]. During the early stage of PPR vaccine virus inoculation in goat PBMC in *vitro*, the GO enrichment analysis revealed that the functions of potential target genes for the DEmiRNAs were mainly to respond against stimuli, biological regulation and immune system process [28]. In comparison with PPR vaccine virus, the responses in sheep or goat infected with PPR virulent virus were different. For PPR virulent virus infection goat PBMC in vivo at 9 dpi, miR-484 which mainly played role in antiviral activities were up-regulated, while miR-30b that inhibited presentation by primary macrophages and antigen processing and miR-21-5p that decreases the sensitivity to IFNs were both down-regulated. In addition, miR-210 which could inhibit apoptosis was down-regulated. The GO enrichment analysis revealed that the function of potential target genes for the DEmiRNAs were significantly enriched in immunological processes including natural killer (NK) T cell differentiation lymphocyte mediated immunity, immunoglobulin mediated immune response, positive regulation of gamma-delta T cell activation, adaptive immune response, regulation of leukocyte differentiation and T cell differentiation [29]. In case of PPR virulent virus infection of sheep and goat in vivo at 10 dpi, the pathogenesis of PPR virulent virus in the spleen and lung of sheep was probably enhanced by miR-320a, miR-363, and miR-21-3p via down-regulating several immune response genes. The highly enriched common GO terms targeted by the DEmiRNAs in the lung tissue of sheep and goats includes TLR6 signaling pathway, T cell receptor signaling pathway and Rap1 signaling pathway [27]. All in all, compared with PPR vaccine virus, the PPR virulent virus might cause a stronger host response, and the DEmiRNAs might play important role in the immune response. In addition, for the known miRNA, miR-744 and miR-574 were the only two common DEmiRNAs special for PPR virulent virus, but not for PPR vaccine virus. Previous reports showed that miR-744 and miR-574 played role in inhibition of cell proliferation and invasion or tumor-suppressive in different human malignancies, which might be novel and efficient therapeutic targets [80,81,82]. In addition, miR-574 also could inhibit apoptosis [83]. On comparing PPR vaccine virus and PPR virulent virus, the miR-30 and let-7 family were commonly differentially expressed in spleen and lung tissues of goats infected with PPR virulent virus in vivo at 10 dpi, PBMC of goat infected with PPR virulent virus in vivo at 9 dpi, PBMC of goat inoculated with PPR vaccine virus in vitro at 1 dpi, and PBMC of sheep inoculated with PPR vaccine virus in *vivo*. The miR-21 was commonly differentially expressed in spleen and lung tissues of goats infected with PPR virulent virus in vivo at 10 dpi, PBMC of goat infected with PPR virulent virus in *vivo*, and PBMC of sheep inoculated with PPR vaccine virus in vivo [27,28,29].

In summary, deep sequencing was used to survey DEmiRNA in sheep PBMC and ST cells inoculated with PPR vaccine virus via time-course. Our researches supplied a series of known and novel DEmiRNAs during PPR vaccine virus inoculation. We identified some crucial miRNAs related to PPR vaccine virus inoculation including the miRNA which were commonly expressed in sheep PBMC cells and ST cells. The dynamic profiles and the regulatory roles of miRNAs via time-course during PPR vaccine virus in sheep could be explored further using the identified miRNAs in our study.

## Figures and Tables

**Table 1:** Primers used for detection of microRNA (miRNA) expression using stem-loop real-time quantitative PCR (RT-qPCR).

miRNA Name	Primer	Sequences
oar-miR-150	RT	GTCGTATCCAGTGCAGGGTCCGAGGTATTCGCACTGGATACGACcactgg
	AS	TCTCCCAACCCTTGTACCAGT
14_7829-5p	RT	GTCGTATCCAGTGCAGGGTCCGAGGTATTCGCACTGGATACGACactaca
	AS	ATTTCCCTGTCTTCAATCCTGTAGT
1_1473-5p	RT	GTCGTATCCAGTGCAGGGTCCGAGGTATTCGCACTGGATACGACagccca
	AS	CGCAAAGAATTCTCCTTTTGG
1_1964-3p	RT	GTCGTATCCAGTGCAGGGTCCGAGGTATTCGCACTGGATACGACccttag
	AS	TAGTCAGGATGGCCGAGCG
21_16124-3p	RT	GTCGTATCCAGTGCAGGGTCCGAGGTATTCGCACTGGATACGACgctcag
	AS	GATCAGTGATGAAAGTAGCCAAATC
5_23932-5p	RT	GTCGTATCCAGTGCAGGGTCCGAGGTATTCGCACTGGATACGACaaccta
	AS	GTCGCTGAGAACTGAATTCCATAG
JH922317.1_29647-5p	RT	GTCGTATCCAGTGCAGGGTCCGAGGTATTCGCACTGGATACGACactcag
	AS	CCGAACAATATCCTGGTGCTG
9_27501-3p	RT	GTCGTATCCAGTGCAGGGTCCGAGGTATTCGCACTGGATACGACcctcaa
	AS	GCTGTCTAGACTGAAGCTCCTTG
1_2854-3p	RT	GTCGTATCCAGTGCAGGGTCCGAGGTATTCGCACTGGATACGACgcctta
	AS	GTCAGGATGGCCGAGCGGT
22_22194-5p	RT	GTCGTATCCAGTGCAGGGTCCGAGGTATTCGCACTGGATACGACacagcc
	AS	CGTGAGAACTGAATTCCATAGGCT
X_39234-3p	RT	GTCGTATCCAGTGCAGGGTCCGAGGTATTCGCACTGGATACGACtatagc
	AS	GTAAAACGTGAGGCGCTGCT
23_22843-3p	RT	GTCGTATCCAGTGCAGGGTCCGAGGTATTCGCACTGGATACGACccggct
	AS	TCGTGTCTTGTGTTGCAGCC
SNORA58	F	CACCTTGCTATGTCTTGGCTTG
	R	ATTCTGCTGGCTGCATCTGAC

**Table 2:** Up/down-regulated miRNAs in sheep PBMC and ST cells.

#of miRNA Changed after PPR Vaccine virus Inoculation							
		Up-Regulated			Down-Regulated		
		3dpi vs. 0dpi	5dpi vs. 0dpi	5dpi vs. 3dpi	3dpi vs. 0dpi	5dpi vs. 0dpi	5dpi vs. 3dpi
PBMC	Known miRNA	8	1	20	21	16	15
	Novel miRNA	167	11	296	177	87	244
ST	Known miRNA	1			2		
	Novel miRNA	108			25		

Differential expressed: log 2 FC ≥ 1 and *p* value ≤ 0.05.

## Supplementary Materials

The following are available online at https://www.mdpi.com/1999-4915/11/11/1025/s1. Table S1. Summary of deep sequencing data of each library are shown in the table. File S1: Differentially expressed known and novel miRNAs at mock and two different time points of PPR vaccine virus inoculated PBMC cell samples, File S2: Differentially expressed known and novel miRNAs in mock and PPR vaccine virus inoculated ST cell samples, File S3: Target genes for differentially expressed known and novel miRNAs at mock and two different time points of PPR vaccine virus inoculated PBMC cell samples, File S4: Target genes for differentially expressed known and novel miRNAs in mock and PPR vaccine virus inoculated ST cell samples, File S5: GO annotations for the predicted target genes for known and novel miRNAs at mock and two different time points of PPR vaccine virus inoculated PBMC cell samples, File S6: GO annotations for the predicted target genes for known and novel miRNAs in mock and PPR vaccine virus inoculated ST cell samples, File S7: KEGG Pathway annotations for the predicted target genes for differentially expressed known and novel miRNAs at mock and two different time points of PPR vaccine virus inoculated PBMC cell samples, File S8: KEGG Pathway annotations for the predicted target genes for differentially expressed known and novel miRNAs in mock and PPR vaccine virus inoculated ST cell samples
