# Supplementary material for: MicroRNA Expression Profile in Peripheral Blood Lymphocytes of Sheep Vaccinated with Nigeria 75/1 Peste Des Petits Ruminants Virus
Source: Viruses. 2019 Nov 5;11(11):1025. doi: 10.3390/v11111025 (PMC6893480; doi:10.3390/v11111025)
Supplement: Supplementary file 1 [file viruses-11-01025-s001.zip › Table S1.docx]

**Table S1.** Summary of deep sequencing data of each library are shown in the table.

| **Library#** | **cells** | **inoculated or mock-** **inoculated** | **Time**  **point** | **unique**  **reads** | **total reads** | **mapped**  **unique reads** | **mapped**  **total reads** |
| --- | --- | --- | --- | --- | --- | --- | --- |
| 1 | PBMC | Mock-inoculated 4 | 0 dpi | 733826 | 10204213 | 93484 | 6117715 |
| 2 | PBMC | Mock-inoculated 5 | 0 dpi | 677122 | 8728631 | 102837 | 5392668 |
| 3 | PBMC | Mock-inoculated 6 | 0 dpi | 389702 | 10562737 | 137709 | 7644332 |
| Mean | | | | 600216 | 9831860 | 111343 | 6384905 |
| 4 | PBMC | PPR vaccine virus-inoculated 4 | 3 dpi | 351337 | 5882470 | 90490 | 3157887 |
| 5 | PBMC | PPR vaccine virus-inoculated 5 | 3 dpi | 398141 | 7899602 | 106039 | 4782988 |
| 6 | PBMC | PPR vaccine virus-inoculated 6 | 3 dpi | 392804 | 8669348 | 100851 | 5357073 |
| Mean | | | | 380761 | 7483807 | 99127 | 4432649 |
| 7 | PBMC | PPR vaccine virus-inoculated 7 | 5 dpi | 593179 | 8403362 | 503438 | 3511102 |
| 8 | PBMC | PPR vaccine virus-inoculated 8 | 5 dpi | 444018 | 7300354 | 67840 | 4475466 |
| 9 | PBMC | PPR vaccine virus-inoculated 9 | 5 dpi | 601447 | 12899616 | 111525 | 8446989 |
| Mean | | | | 546215 | 9534444 | 227601 | 5477852 |
| 10 | ST | Mock-inoculated 1 | 3 dpi | 207678 | 8979911 | 46011 | 6363030 |
| 11 | ST | Mock-inoculated 2 | 3 dpi | 212765 | 9105583 | 46663 | 6499084 |
| 12 | ST | Mock-inoculated 3 | 3 dpi | 220558 | 9778943 | 48699 | 6824731 |
| Mean | | | | 213667 | 9288146 | 47124 | 6562282 |
| 13 | ST | PPR vaccine virus-inoculated 1 | 3 dpi | 728187 | 9464557 | 86926 | 2731715 |
| 14 | ST | PPR vaccine virus-inoculated 2 | 3 dpi | 717210 | 9917314 | 83315 | 3195797 |
| 15 | ST | PPR vaccine virus-inoculated 3 | 3 dpi | 677420 | 9993112 | 77643 | 3519328 |
| Mean | | | | 707606 | 9791661 | 82627 | 3148946 |
